# Intermediate Phase‐Free Process for Methylammonium Lead Iodide Thin Film for High‐Efficiency Perovskite Solar Cells

**DOI:** 10.1002/advs.202102492

**Published:** 2021-09-17

**Authors:** Yeonghun Yun, Devthade Vidyasagar, Minho Lee, Oh Yeong Gong, Jina Jung, Hyun‐Suk Jung, Dong Hoe Kim, Sangwook Lee

**Affiliations:** ^1^ School of Materials Science and Engineering Kyungpook National University Daegu 41566 Republic of Korea; ^2^ School of Advanced Materials Science and Engineering Sungkyunkwan University Suwon 16419 Republic of Korea; ^3^ Department of Nanotechnology & Advanced Materials Engineering Sejong University Seoul 05006 Republic of Korea; ^4^ Department of Materials Science and Engineering Korea University 145 Anam‐ro, Seongbuk‐Gu Seoul 02841 Republic of Korea

**Keywords:** defect density, donor number, intermediate phase, Lewis base, perovskite solar cells

## Abstract

Solvent engineering by Lewis‐base solvent and anti‐solvent is well known for forming uniform and stable perovskite thin films. The perovskite phase crystallizes from an intermediate Lewis‐adduct upon annealing‐induced crystallization. Herein, it is explored the effects of trimethyl phosphate (TMP), as a novel aprotic Lewis‐base solvent with a low donor number for the perovskite film formation and photovoltaic characteristics of perovskite solar cells (PSCs). As compared to dimethylsulfoxide (DMSO) or dimethylformamide (DMF), the usage of TMP directly crystallizes the perovskite phase, i.e., reduces the intermediate phase to a negligible degree, right after the spin‐coating, owing to the high miscibility of TMP with the anti‐solvent and weak bonding in the Lewis adduct. Interestingly, the PSCs based on methylammonium lead iodide (MAPbI_3_) derived from TMP/DMF‐mixed solvent exhibit a higher average power conversion efficiency of 19.68% (the best: 20.02%) with a smaller hysteresis in the current‐voltage curve, compared to the PSCs that are fabricated using DMSO/DMF‐mixed (19.14%) or DMF‐only (18.55%) solvents. The superior photovoltaic properties are attributed to the lower defect density of the TMP/DMF‐derived perovskite film. The results indicate that a high‐performance PSC can be achieved by combining a weak Lewis base with a well‐established solvent engineering process.

## Graphical abstract

A low defect halide perovskite film can be fabricated using trimethyl phosphate (TMP). TMP directly forms perovskite without intermediate phase due to weak Lewis basicity. The TMP‐based film reduces hysteresis in current‐voltage curve of perovskite solar cell. The TMP‐based device exhibits better performance (>20%) than other solvent‐based ones.

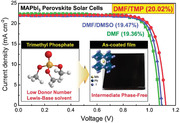

## 1 Introduction

Organic‐inorganic halide perovskite solar cells (PSCs) have made remarkable progress as next‐generation photovoltaics. Over the past decade, the certified power conversion efficiency (PCE) of single‐junction PSCs has sharply increased to 25.5%.^[^ ^1^ ^]^ The rapid improvement in PCE of PSCs is mainly accompanied by the advances in fabrication process such as solvent engineering strategy,^[^ ^2^ ^]^ as well as the developments in desirable composition of light absorptionmaterial such as mixed‐halide and mixed cation perovskites,^[^ ^3^ ^]^ and device configuration with new charge transport layers.^[^ ^4^ ^]^

Solvent engineering is the most commonly used and efficient method for the fabrication of high‐quality perovskite (PVSK) layer. This method is implemented by spin‐coating a PVSK precursor solution that is based on polar aprotic solvents, such as dimethylformamide (DMF) and dimethyl sulfoxide (DMSO), and followed by dripping a non‐polar anti‐solvent before the end of the spin‐coating.^[^ ^2a,b^ ^]^ The anti‐solvent dripping process reduces the solubility of the PVSK salts by eliminating the miscible solvents. Therefore, an intermediate phase can be achieved by solidification and crystallization of the Lewis adduct, which is a coordination compound of a Lewis acid (e.g., PbI_2_) and a Lewis base (e.g., DMSO) in the PVSK precursor.^[^ ^5^ ^]^

The formation of the intermediate phase largely depends on the basicity of the Lewis base. When a strong Lewis base such as DMSO is used, only the intermediate phase without PVSK is produced after dripping of an anti‐solvent, due to high polarity of the Lewis base and thus low miscibility with the anti‐solvent. In general, the intermediate phase film is transformed to a PVSK film via a post‐annealing at around 100–150 °C. During the annealing, dissociation and vaporization of the Lewis base result in nucleation of PVSK crystals and simultaneous grain growth thereof. There have been many studies based on this strategy, reporting the formation of high‐quality PVSK films comprised of relatively uniform and large grains with enhanced reproducibility.^[^ ^5^, ^6^ ^]^ It is understood that the intermediate phase based on strongly bonded Lewis adduct suppresses irregular rapid nucleation and abnormal grain growth.^[^ ^7^ ^]^

On the other hand, PVSK and the intermediate phase can coexist in a film after the anti‐solvent dripping, depending on the basicity of the Lewis base. Using a weak Lewis base, the intermediate phase can be reduced or even totally removed after the anti‐solvent dripping, owing to their high solubility with an anti‐solvent.^[^ ^8^ ^]^ In fact, the formation of the intermediate phase is not essential for a high‐quality PVSK film, if a uniform film based on a large population of uniform PVSK nuclei can be achieved by the spin‐coating process. If the Lewis base could be removed by the anti‐solvent dripping, direct nucleation of PVSK can be induced by the spin‐coating, hence the post‐annealing induces only the grain growth. In this case, concurrent annealing leads to less volume change, thus short‐range mass diffusion. However, as per the author's knowledge, there are limited reports on high‐efficiency PSCs using a low donor number Lewis‐base solvent.

In this paper, we investigated the effects of weak Lewis‐base solvent on the perovskite film formation and photovoltaic properties of PSCs. A high‐quality methylammonium lead iodide (MAPbI_3_) film was fabricated using trimethyl phosphate (TMP) as a novel, low donor number Lewis‐base. A negligible amount of intermediate phase was observed after anti‐solvent dripping, due to a high solubility of TMP with the anti‐solvent. It is clear that the addition of a small amount of TMP to DMF results in the direct formation of PVSK film without any so‐called intermediate phase in as‐coated film. Interestingly, the PSCs, fabricated based on the MAPbI_3_ film derived from TMP/DMF‐mixed solvent in ambient air, exhibited superior photovoltaic properties (the best PCE of 20.02%) with a higher open‐circuit voltage (*V*_OC_) and a smaller hysteresis in the current‐voltage curve, compared to the PSCs fabricated using DMSO/DMF‐mixed or DMF‐only solvents. The high *V*_OC_ and the small hysteresis were attributed to a low defect density of the TMP/DMF‐derived PVSK film. Given that the reported best PCE of MAPbI_3_‐based PSCs is 21.90% to our knowledge,^[^ ^9^ ^]^ our finding suggests that a high‐performance PSC can be achieved by combining a weak Lewis base with a well‐established solvent engineering process.

## 2 Results and Discussion

The molecular structure and donor number in **Table** **1** indicate the degree of Lewis basicity of polar aprotic solvents DMSO, DMF, and TMP.^[^ ^10^ ^]^ All three solvents could be depicted as O‐donor Lewis bases owing to the lone‐pair electrons of the oxygen atoms. The donor number decreases with varying the bonding counter element, sulfur (DMSO), carbon (DMF), and phosphorus (TMP). A solvent having much lower polarity was not considered, because it is not able to strongly coordinate with PVSK salts, and thus solubility of PVSK salts in it will be too low to produce high‐quality PVSK films. The strength of binding energy between PVSK salt and the solvent is known to have a significant impact on the formation of a stable intermediate phase, which affects the growth of polycrystalline PVSK films.^[^ ^6^, ^11^ ^]^

**Table 1:** Molecular structures and donor numbers of each solvent^[^ ^10^ ^]^

	DMSO (Dimethyl sulfoxide)	DMF (Dimethylformamide)	TMP (Trimethyl phosphate)
Molecular structure	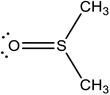	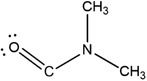	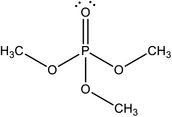
Donor number	29.8	26.6	23.0

**Figure 1:**
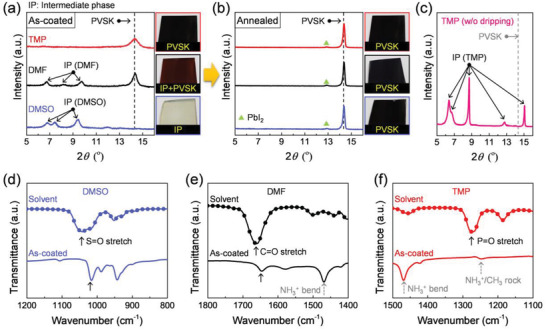
Characteristics of spin‐coated thin films based on the mono‐solvents. a,b) XRD and photo images of a) as‐coated and b) annealed perovskite films obtained from each solvent (DMSO, DMF, and TMP). c) XRD pattern of TMP‐based as‐coated film without anti‐solvent dripping during solvent engineering. Herein, the intermediate phase (IP) derived from DMSO, DMF, and TMP were indicated as IP (DMSO), IP (DMF), and IP (TMP), respectively. d–f) FTIR spectra of d) DMSO, e) DMF, and f) TMP with each mono‐solvent‐based as‐coated films. The arrows indicate the representative peak of each solvent.

**Figure** **1** shows the material properties of the as‐coated and annealed MAPbI_3_ PVSK films using DMF, DMSO, and TMP mono‐solvents. All the films were fabricated via solvent engineering method with anti‐solvent diethyl ether (DEE) dripping. The XRD patterns and digital photo images of as‐coated PVSK films show different diffraction peaks and appearance colors, depending on the precursor solvent (Figure 1a). The DMSO‐based as‐coated film is transparent and changes to opaque black film after the annealing (Figure 1b). The DMSO‐based as‐coated film shows only the DMSO‐incorporated intermediate phase, evidenced by the XRD peaks at 6.8°, 7.4°, and 9.4°.^[^ ^2^, ^12^ ^]^ PVSK‐related XRD peaks are not observed at all. The DMF‐based film appears to be brown before the annealing and black after the annealing. From the XRD pattern of the DMF‐based as‐coated film, the peaks corresponding to PVSK (14.3^o^) and DMF‐incorporated intermediate phase (6.7°, 8.2°, and 9.7°)^[^ ^12^ ^]^ are observed. In stark contrast, the TMP‐based as‐coated film is already black, and the XRD pattern shows only PVSK peaks without any peaks for the secondary phase, indicating negligible TMP‐incorporated intermediate phase in the film. The absence of the intermediate phase is most likely due to extraction of TMP during the anti‐solvent dripping, because of the high miscibility of TMP with DEE and the weak binding between TMP and the Lewis acid molecules. It should be noted that without anti‐solvent dripping TMP forms the intermediate phase film without PVSK, evidenced by the non‐perovskite XRD peaks at low 2*θ* region (Figure 1c). After thermal annealing, all the films transform to the PVSK phase, as shown in Figure 1b.

The existence of the intermediate phase in each film was further analyzed by comparing the FTIR spectrum of each solvent and as‐coated film (Figure 1d–f). The stretching vibration of S═O appears at 1045 cm^−1^ for DMSO and shifts to 1016 cm^−1^ for the DMSO‐based as‐coated film.^[^ ^6^, ^7^ ^]^ Similarly, the C═O stretching peak of DMF at 1665 cm^−1^ is shifted to 1647 cm^−1^ for the DMF‐based as‐coated film.^[^ ^13^ ^]^ The downshift of S═O and C═O stretching peak indicates the formation of the intermediate phase caused by the interaction between the solvents and salts. However, in the case of the TMP‐based as‐coated film, the P═O stretching peak is not observed at around 1276 cm^−1^.^[^ ^14^ ^]^ The small peak at 1252 cm^–1^ is corresponding to NH_3_^+^/CH_3_ rocking vibration modes of MA, not P═O stretching, as it is observed from all other annealed MAPbI_3_ films using other solvents (Figure S1a, Supporting Information).^[^ ^15^ ^]^ After the annealing process, all the films exhibit only the MAPbI_3_ peaks without any solvent peaks, regardless of the solvents (Figure S1b, Supporting Information).^[^ ^16^ ^]^

**Figure 2:**
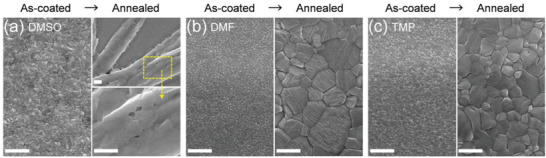
Morphology evolution of mono‐solvent‐based films. a–c) SEM images of as‐coated and annealed perovskite films based on a) DMSO, b) DMF, and c) TMP. Scale bars: 500 nm.

The different states of each as‐coated film have caused a great difference in the morphology of annealed PVSK films. **Figure** **2**a–c presents the comparison of the morphologies of mono‐solvent‐based as‐coated and annealed PVSK films. We note that the SEM image of the as‐coated DMSO and DMF films can be slightly different from a real morphology, since the vacuum induces growth of the intermediate grains and phase transformation from the intermediate to the perovskite to some degree.^[^ ^17^ ^]^ However, no significant difference is observed for all the three kinds of solvent based as‐coated films. In contrast, after annealing, DMSO exhibits a needlelike macro‐structure with inhomogeneous polycrystal grains, whereas DMF and TMP exhibit relatively dense and uniform grains. Therefore, from the XRD and SEM analyses, it can be understood that the donor number influences the route for crystallization of perovskite. In the case of high donor number, perovskite precursor sequentially forms adduct, intermediate phase film, and simultaneous nucleation and growth of perovskite crystallites in the film. In contrast, in the case of low donor number, perovskite precursor sequentially forms adduct, perovskite crystallite film, and then growth of perovskite crystallites in the film.

It is notable that the DMSO‐based annealed film shows agglomerated morphology which is significantly different compared to the other annealed films. We attribute the different morphology of the DMSO‐based annealed film to a large amount of residual DMSO in the as‐coated film. DMF and TMP are miscible with DEE, whereas DMSO has a very low solubility in DEE. Therefore, after the DEE dripping process (during the spin‐coating), most of DMF and TMP solvents are washed out, while DMSO is not. During the annealing process, evaporation of the solvents occurs, and thus, a large amount of residual DMSO retards volatilization rate of the solvent, which can induce formation of agglomerates. Aside from the ability of DMSO to significantly improve process stability and film quality, DMSO mono‐solvent generates irregular grains and unwanted defects during crystallization because of the strong interaction between the salt and solvent. For this reason, DMSO is generally used with DMF as a form of mixed solvent for precursor solution.

**Figure 3:**
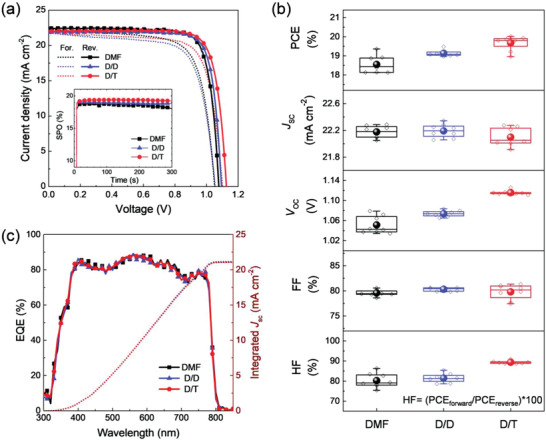
Photovoltaic performance of perovskite solar cells based on different solvents (DMF, D/D, and D/T). a) *J*–*V* curves of the best‐performing devices. The inset indicates the stabilized power output of the corresponding devices. b) Statics of PCE, *J*_SC_, *V*_OC_, FF, and HF obtained from perovskite solar cells with different solvents. HF is calculated using the equation HF = (PCE_forward_/PCE_reverse_)*100. c) Wavelength‐dependent EQE and integrated *J*_SC_ of devices fabricated using different solvents.

To confirm the impact of Lewis basicity of solvents on the PVSK quality and their photovoltaic properties, we fabricated the PVSK films using different mixed‐solvent systems: pure DMF (control), DMF/1.0 m DMSO (hereafter simplified as D/D), and DMF/1.0 m TMP (D/T). The solar cell devices that were fabricated using D/T‐based solvents exhibited the most excellent photovoltaic properties compared with the mono‐DMF‐ and the D/D‐based devices. **Figure** **3**a and the inset present the *J*–*V* curves and the stabilized power output (SPO) of the best‐performing devices. The best PCE (and SPO) of each sample is 19.36% (18.82%), 19.47% (19.10), and 20.02% (19.40) for the DMF‐, D/D‐, and D/T‐based device, respectively. Figure 3b and **Table** **2** present the statistical photovoltaic parameters of the three kinds of the solvent system. The D/T‐based devices exhibit an average PCE of 19.68% ± 0.35%, with the average short‐circuit current density (*J*_SC_), open‐circuit voltage (*V*_OC_), and fill factor (FF) of 22.10 ± 0.12 mA cm^−2^, 1.116 ± 0.004 V, and 79.79% ± 1.41%, respectively. It is evident that the average *V*_OC_ of the D/T‐based devices is improved, compared to that of the DMF‐based (1.051 ± 0.017 V) and D/D‐based (1.074 ± 0.006 V) devices, whereas the *J*_SC_ and FF are similar for all. Furthermore, the *J*–*V* hysteresis factor (HF, defined in Figure 3b) is significantly improved in the D/T‐based devices. The average HF of D/T is 89.39% ± 0.677%, which is about 10% higher than those of the DMF‐based (80.24% ± 3.34%) and D/D‐based (81.47% ± 2.13%) devices. In general, the origin of hysteresis in the PSCs is known as the ion migration, charge trapping at interfaces and grain boundary, and ferroelectric effects of PVSK.^[^ ^18^ ^]^ Particularly, the defects in the film can act as trap sites and consequently derive the capacitance related to the accumulation of the interfacial charge and/or photogenerated charge carrier trapping, which leads to a large *J–V* hysteresis.^[^ ^19^ ^]^ Thus, the different qualities of PVSK derived from solvents are believed to significantly influence the HF. The device performance such as *V*_OC_ and HF improves as the TMP/perovskite ratio approaches 1.0 and then saturates, similar to change in uniformity of grain size, as shown in Figures S2, S3 and Table S1 (Supporting Information). The integrated *J*_SC_s calculated from EQE were 21.16, 21.23, and 21.11 mA cm^−2^ for the DMF‐, D/D‐, and D/T‐based devices (Figure 3c). These values were found to match well with the *J*_SC_s of the *J*–*V* scan, regardless of the solvent type. It is consistent that the added solvents did not affect their thickness and absorbance (Figures S4 and S5, Supporting Information).

**Table 2:** Best and average metrics (PCE, *J*_SC_, *V*_OC_, FF, and HF) of perovskite solar cells fabricated using the DMF, D/D, and D/T solvents

		PCE [%]	*J*_SC_ [mA cm^−2^]	*V*_OC_ [V]	FF [%]	HF [%]
DMF	Best	19.36	22.28	1.079	80.55	83.47
	Average	18.55 ± 0.42	22.18 ± 0.08	1.051 ± 0.017	79.57 ± 0.560	80.24 ± 3.34
D/D	Best	19.47	22.28	1.083	80.69	80.90
	Average	19.14 ± 0.15	22.19 ± 0.09	1.074 ± 0.006	80.31 ± 0.336	81.47 ± 2.13
D/T	Best	20.02	22.07	1.126	80.55	89.51
	Average	19.68 ± 0.35	22.10 ± 0.12	1.116 ± 0.004	79.79 ± 1.41	89.39 ± 0.677

**Figure 4:**
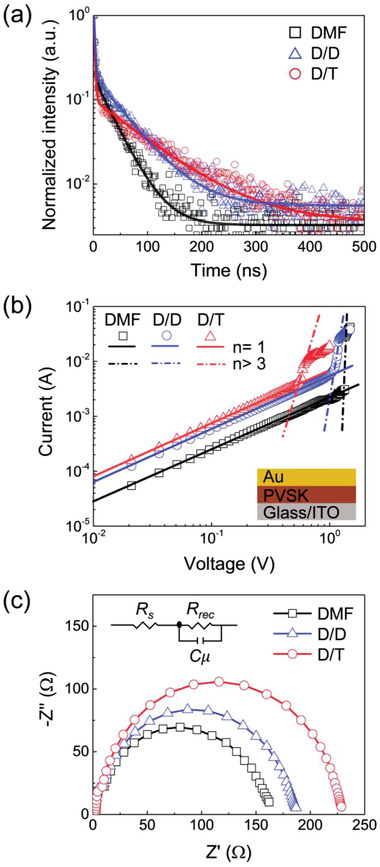
Lifetime of photogenerated charge carriers, SCLC, and EIS analysis of annealed perovskite films obtained from the DMF, D/D, and D/T solvents. a) Normalized time‐resolved photoluminescence decay profiles for glass/ITO/SnO_2_/PVSK films.^[^ ^20^ ^]^ b) Dark current‐voltage curves for glass/ITO/PVSK/Au films. Solid and dash‐dot lines indicate the regions with a slope of 1 and >3, respectively.^[^ ^21^ ^]^ c) Nyquist plots of the perovskite solar cells fabricated using the DMF, D/D, and D/T solvents. EIS measurements were conducted under dark conditions.^[^ ^20^, ^22^ ^]^

To reveal the reason for the enhanced *V*_OC_ and HF of the D/T‐based PVSK film, the defect density of the bulk PVSK and interface between the charge transport layers (CTL) and PVSK layer were investigated. The TRPL spectra of glass/ITO/SnO_2_/PVSK films were measured to evaluate the lifetime of photogenerated charge carriers (**Figure** **4**a).^[^ ^20^ ^]^ All the PVSK films with different solvents exhibit biexponential decay with fast (*τ*_1_) and slow (*τ*_2_) characteristic time. **Table** **3** presents the fitted parameters. Commonly, *τ*_1_ is related to the quenching of charge carriers at the interface by extracting them from PVSK film to CTL, whereas *τ*_2_ is associated with the radiative recombination of the generated free charge carriers due to the traps in the PVSK.^[^ ^23^ ^]^ The extracted *τ*_1_ of the PVSK films based on the D/T precursor is very slightly faster (<≈10%) than those of the DMF and the D/D precursor solutions. In fact, the difference in *τ*_1_ between DMF‐, D/D‐, and D/T‐based films are as small as 2.069 ±  0.115 that only ± 5.5% differences, indicating comparable charge extraction among the samples. In stark contrast, *τ*_2_ of the DMF‐, D/D‐, and D/T‐based PVSK films are significantly different from each other; 33.74, 55.90, and 103.4 ns, respectively. We note that the slightly smaller *τ*_ave_ of the D/T‐based film is resulted from the fast *τ*_1_, because the *τ*_ave_ is calculated from the amplitude‐weighted average lifetime. The device structure with CTL usually has much higher contribution of *τ*_1_ than *τ*_2_, *τ*_ave_ is highly affected by the *τ*_1_. The prolonged lifetime of the charge carriers indicates the suppressed defect density of the PVSK layer, which results in the improved *V*_OC_ and HF characteristics of D/T‐based PSCs.

**Table 3:** Calculated charge carrier lifetime of the perovskite films fabricated using the DMF, D/D, and D/T solvents

	A1 [%]	*τ*_1_ [ns]	A2 [%]	*τ*_2_ [ns]	*τ*_average_ [ns]
DMF	99.99	2.041	0.010	33.74	2.04
D/D	87.61	2.223	12.39	55.90	8.88
D/T	94.47	1.945	5.530	103.4	7.56

Further direct analysis of the defect density of the PVSK film was conducted using the dark *I*–*V* characteristics, as presented in Figure 4b. The *I*–*V* curve can be divided into three regions: ohmic linear region at a low bias voltage of *I*∝*V* ^*n*  =  1^, nonlinear trap‐filled limit (TFL) region of *I*∝*V* ^*n* > 3^, and the space charge‐limited‐current (SCLC) region of *I*∝*V* ^*n*  =  2^. The TFL voltage (*V*_TFL_) is extracted from the intersection of the ohmic and TFL regions, and the trap density, *n*_t_, is calculated using the following equation:^[^ ^21^ ^]^

(1) $$\;V_{\mathrm{TFL}}=\frac{en_{t}L^{2}}{2\varepsilon \varepsilon_{0}}\;$$

where *e* denotes elementary charge, *L* denotes the thickness of the PVSK film (480 nm), *ε* denotes the relative dielectric constant of MAPbI_3_ (28.8),^[^ ^24^ ^]^ and *ε*_0_ denotes the vacuum permittivity (8.854  ×  10^−14^ F cm^−1^). The calculated trap density of the PVSK layer is 1.8  ×  10^15^, 1.4  ×  10^15^, and 7.6  ×  10^14^ cm^−3^ for the DMF‐, D/D‐, and D/T‐based PVSK films, respectively. Similar to the trends of TRPL, the trap density of the D/T‐based PVSK film is significantly less than those of the DMF‐ and D/D‐based films. This result supports that the enhanced *V*_OC_ and HF of the D/T‐based PSCs are derived from the low defect density of the PVSK, thus prolonged the lifetime of the charge carriers.

The defect density between CTL and PVSK was also investigated by EIS.^[^ ^20^, ^22^ ^]^ Figure 4c presents the Nyquist plots of the devices, by which the series resistance (*R*_s_) and recombination resistance (*R*_rec_) in parallel with the chemical capacitance (*C_*μ*_*) at the interface could be extracted. As no significant difference was observed in the fitted *R_s_* for each solvent type, the properties of the independent layers were expected to be similar. However, extracted *R*_rec_ are different, i.e., 162, 187, and 229 Ω for the DMF‐, D/D‐, and D/T‐based PSCs, respectively. In general, the higher *R*_rec_ value rendered the charge recombination difficult.^[^ ^22^ ^]^ Thus, the highest *R*_rec_ value of the D/T‐based PSCs had the most suppressed charge recombination among the perovskite grains, which led to the enhancement of the *V*_OC_ of PSCs.^[^ ^25^ ^]^

**Figure 5:**
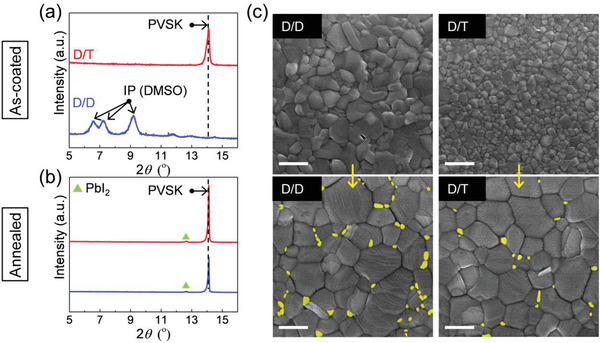
Characteristics of the thin films based on the D/D and D/T solvents. a,b) XRD patterns of a) as‐coated and b) annealed perovskite films fabricated using the D/D and D/T solvents. IP (DMSO) is the DMSO‐incorporating intermediate phase described in Figure 1a. c) SEM images showing the morphology change of as‐coated and annealed perovskite films obtained from the D/D and D/T solvents (Scale bars: 500 nm). The grains colored in yellow indicate the small sized (<50 nm) grains along the grain boundaries.

To determine the reason why D/T‐based PVSK film has a low defect density, we took a closer look at how TMP affects the transition from precursor solution to PVSK. When the equivalent molar ratio of the TMP to the MAPbI_3_ concentration is added, the as‐coated film is composed of only the PVSK phase without any DMF‐incorporated intermediate phase (**Figure** **5**a). The intermediate phase gradually diminished with the increase in the ratio of TMP to PVSK (Figure S6, Supporting Information). Therefore, it is evident that a small amount of TMP hindered the formation of the intermediate phase. However, the D/D as‐coated film has a DMSO‐incorporated intermediate phase similar to that of the DMSO mono‐solvent‐based film. This is also confirmed in the FTIR spectra presented in Figure S7 (Supporting Information), which demonstrates the absence of the P═O peak and presence of the S═O peak in the D/T‐ and D/D‐based films, respectively. The absence of P species was double‐checked with x‐ray photoelectron spectroscopy, as shown in Figure S8 (Supporting Information).

Because of the low solubility of DMSO in DEE, it is reasonable that DMSO and D/D solvents form the DMSO‐related intermediate phase after dripping. On the other hands, in the case of DMF, TMP and D/T solvents, corresponding intermediate phases can be made or not, depending on i) the bonding force of adduct, and ii) the dripping amount of DEE. The bonding between DMF and salts is stronger than that between TMP and salts, much larger amount of DEE is needed to fully dissociate the DMF from the DMF‐adduct, compared to the TMP‐adduct, as shown in Figure S9a (Supporting Information). Similarly, we found that the as‐coated D/T film also has the IP (DMF) with a low amount of DEE dripping and different dripping timing, as shown in Figure S9b,c (Supporting Information). Therefore, it can be concluded that pouring sufficiently large amount of anti‐solvent can lead to an intermediate phase‐free film even in the case of DMF. However, it is notable that excessive washing with anti‐solvent leads to loss in materials for film formation and the resultant film can have poor morphology and bad device performance. Furthermore, different dripping amount and timing create too many variables for film quality, which makes it difficult to understand the effect of the donor number of solvents and the absence of intermediate phase.

We also suspected presence of amorphous in the as‐coated D/T‐based film. However, Figure S10 (Supporting Information) shows that there is no significant change in the base line before and after the annealing, indicating negligible amorphous in the as‐coated D/T‐based PVSK film. Therefore, we attribute the increase in peak intensity and the decrease in full width at half maximum to crystal growth by Ostwald ripening,^[^ ^26^ ^]^ as evidenced by the evolution of grains from dense, small grains to dense, large grains, as shown in Figure 5c. Another reason of the peak increase and the sharpening is the preferential orientation of the grains after annealing. The main peak for (110) plane significantly increased by the annealing while the peak for the (204)/(312) planes did not, as shown in Figure S10 (Supporting Information).

Based on the above investigation, it can be concluded that the D/T‐based film undergoes i) nucleation of PVSK from adduct during the spin‐coating process and ii) grain growth during the annealing process. In this case, the generation of undesired defects could be suppressed due to small volume change during the annealing process. When the DMSO‐incorporated intermediate phase is present in the as‐coated film, a large volume change of as much as 15% occurs during the phase transition due to the difference in density between the intermediate phase (3.61 g cm^−3^) and MAPbI_3_ (4.16 g cm^−3^).^[^ ^27^ ^]^ This volume change is likely to cause a long‐range diffusion of elements during filling out the empty space by the densification, which can induce defects and negative effects to the final products. The excellent crystallinity of the D/T‐based PVSK shown in Figure 5b supports the explanation; the (110) XRD peak intensity of the D/T‐based PVSK at around 14° is 1.6 times higher than that of the D/D‐based PVSK. Furthermore, the wide range XRD patterns (Figure S11, Supporting Information) of the D/D‐and the D/T‐based PVSK films show (110)‐preferred orientation of the grains in the D/T‐based film. The preferential grain growth along the (110) plane results in surficial homogeneity within facets, which is well‐known to improve the photovoltaic performances of PSCs.^[^ ^28^ ^]^ It is reported that the photovoltaic property of individual perovskite grain is directly influenced by the crystal facet of PVSK, because of the facet dependent density of trap states.^[^ ^29^ ^]^ Therefore, the nature of (110) preferred‐orientation is considered as one of the important reasons for the low defect density of the D/T‐based film.

Figure 5c shows the SEM plan views of both as‐coated and annealed PVSK films using the D/D and D/T solvents. We note again that the SEM images of the as‐coated films are slightly different from real (see Figure S12 and followed discussion in Supporting Information, for more detail). In as‐coated films, the D/T‐based film exhibited smaller and more uniform grains than the D/D‐based as‐coated PVSK film. Small grains of D/T‐based as‐coated PVSK film can facilitate the densification of PVSK with low defect density due to a large number of grain boundaries responsible for the diffusion path of the pores and defects. In the conventional solid‐state‐reaction‐based bulk sintering process, it is well known that sintering of smaller grains accompanying more grain boundaries results in more dense and higher quality polycrystalline films, because the grain boundary is an active transport path for mass transport, according to the concept of Coble creep.^[^ ^30^ ^]^ After annealing, both the PVSK films exhibit similar grain sizes for large grains (a few hundreds of nm), while many small grains of <50 nm are observed at the grain boundaries of the D/D‐derived PVSK films, which also support the suggested explanation.

The atmospheric robustness of the devices was investigated by a moisture stability and a thermal stability tests. At first humidity effect on unencapsulated devices was tracked by storing devices at 40–50% of relative humidity (RH) at 20° C, as shown in Figure S13 (Supporting Information). D/T‐based device maintained 98.4% of its original PCE after 435 h while DMF‐based device drops its PCE quickly to 58.7% after 435 h. D/D‐based device showed slightly lower stability than D/T‐based device by maintaining 96.6% of its pristine PCE. For thermal stability test, devices were continuously heated at 60 °C in N_2_ atmosphere. Similar to the humidity effect, D/T‐based device showed the superior thermal stability by maintaining 96.7% of the original PCE after 435 h. D/D‐ and DMF‐based device retained 93.2% and 81.8%, respectively. The better stability of the D/T‐based device is understandable, since it is reported that a compact uniform PVSK film enhances the stability as well as the photovoltaic performance,^[^ ^31^ ^]^ and a higher density of defects lead poor device stability.^[^ ^32^ ^]^

## 3 Conclusion

In summary, we investigated the effects of TMP as a novel weak Lewis‐base for the formation of PVSK thin film. TMP‐derived PVSK precursor drastically reduced the formation of intermediate phase after anti‐solvent dripping, which enabled direct perovskite grain growth by post‐annealing. Notably, the TMP/DMF‐derived PSCs exhibited an improved PCE of 20.02% with enhanced *V*_OC_ than the DMSO/DMF‐based devices. A significant increase in *V*_OC_ and reduction in *J–V* hysteresis of TMP/DMF‐derived PSCs is ascribed to the low defect density of the PVSK layer. Our results demonstrate that a high‐performance PSC can be achieved using a weak Lewis base by separating the nucleation and grain growth stages in the formation of PVSK film.

## 4 Experimental Methods

### Perovskite Film Coating

PVSK precursor solutions were prepared by dissolving 1.65 m of MAI (Xi'an Polymer Light Technology Corp., ≥99.5%) and 1.65 m of PbI_2_ (Tokyo Chemical Industry, 99.99%) in the mono‐solvent system (DMSO (Sigma‐Aldrich, anhydrous, ≥99.9%), DMF (Sigma‐Aldrich, anhydrous, 99.8%), or TMP (Sigma‐Aldrich, ≥99%)), or mixed‐solvents system (DMF/DMSO (x/x; v/v ratio) or DMF/TMP (x/x; v/v ratio)). Mixed solvents were based on the mono‐DMF solvent, and the amount of DMF proportional to the concentration of MAPbI_3_ was replaced by DMSO or TMP. Spin‐coating was set at 4000 rpm for 20 s (ramp for 3000 rpm s^−1^), and a 0.5 mL of diethyl ether (DEE, Sigma‐Aldrich, anhydrous, ≥ 99.7%) was dropped at 9 s. After spin‐coating, as‐coated PVSK films were sequentially annealed at 65 °C and 130 °C for 1 and 10 min, respectively.

### Device Fabrication

Fabrication and characterization were performed under ambient conditions at a temperature of ≈25 °C and humidity of ≈30%. About 2 × 2 cm^2^ of tin‐doped indium oxide (ITO, Samsung Corning, on Eagle glass, 6–7 Ω sq^−1^) substrates were cleaned with acetone, deionized water, and isopropyl alcohol in an ultrasonic bath for 10 min each. The cleaned substrates were dried under N_2_ gas stream and treated for 20 min under the UV‐O_3_ plasma. Subsequently, 2.5 wt% of SnO_2_ colloidal (Alfa Aesar, 15% in H_2_O colloidal dispersion) solution diluted with deionized water was spin‐coated on the substrates at 3000 rpm for 30 s and dried at 150 °C for 30 min. The PVSK layer was coated, as previously described, and the hole transport layer was coated on the PVSK films by spin‐coating the solution at 3000 rpm for 30 s. The hole transport layer was prepared by dissolving 80 mg of spiro‐OMeTAD (Luminescence Technology Corp., >99.5%), 32 µL of 4‐tertbutylpyridine (Sigma‐Aldrich, anhydrous, 96%), 18 µL of Li‐TFSI solution (566 mg of Li‐TFSI (Alfa Aesar, 99%) dissolved in 1 mL of acetonitrile (Sigma‐Aldrich, anhydrous, 99.8%)), and 14.7 mg of cobalt (III) TFSI (Greatcell Solar) in 1 mL of chlorobenzene (Sigma‐Aldrich, anhydrous, 99.8%). Finally, a 100‐nm‐thick gold electrode was deposited via thermal evaporation.

### Characterization

Crystallographic analysis of as‐coated and annealed PVSK thin films was conducted via X‐ray diffraction (XRD, X'Pert, PANalytical) with CuK*α* rays. Moreover, an infrared spectroscopic study was conducted using Fourier transform‐infrared spectrometer (FTIR, Frontier, PerkinElmer). Particularly, the XRD patterns and FTIR spectra of as‐coated films were obtained as soon as possible after coating. The surface morphology and cross‐sectional images were observed via field‐emission scanning electron microscopy (FE‐SEM, JSM‐6701F, JEOL). For further investigation of the photovoltaic properties of the PSCs, a solar simulator (Oriel Solar 3A Class, 94023A, Newport), which was calibrated to illuminate AM 1.5G (100 mA cm^−2^) using a standard Si reference cell (91150V, Newport), was utilized. The current density–voltage (*J*–*V*) curves of the devices were obtained using a source meter (Keithley 2450, Keithley), from forward bias to reverse bias, at a scan rate of 100 mV s^−1^. The illuminated active area of the device was 0.1 cm^2^. In addition, the stabilized power output (SPO) and external quantum efficiency (EQE) spectra of the PSCs were measured using a potentiostat (PGSTAT204, Autolab) and a tunable light source for QE (TLS‐300XU, Newport), respectively. The absorption spectra of the PVSK thin films were obtained via UV–vis–NIR spectroscopy (Cary 5000, Agilent Technologies). Using photoluminescence spectrometers, the time‐resolved photoluminescence (TRPL) spectra were measured (Quantaurus, Hamamatsu Photonics); the excitation (emission) wavelength was also measured at 464 (770) nm. For the SCLC analysis of glass/ITO/PVSK/Au, the current‐voltage curves were obtained using a mechanical probe station and source meter (Keithley 4200‐SCS, Keithley) under dark conditions. Electrochemical impedance spectra (EIS) were conducted using Keithley 2450 with 1 V biasing under the dark condition to evaluate the effect of background carriers. Energy‐dispersive X‐ray spectroscopy analysis was conducted using HITACHI S‐4800, which was equipped with Horiba EX‐250. The surface chemical states of the PVSK films were analyzed via x‐ray photoelectron spectroscopy (XPS, Theta Probe AR‐XPS System, Thermo Fischer Scientific).

## Conflict of Interest

The authors declare no conflict of interest.

## Supporting information

Supporting Information

## Data Availability

The data that support the findings of this study are available from the corresponding author upon reasonable request.
