# Supplementary material for: Intermediate Phase‐Free Process for Methylammonium Lead Iodide Thin Film for High‐Efficiency Perovskite Solar Cells
Source: Adv Sci (Weinh). 2021 Sep 17;8(21):2102492. doi: 10.1002/advs.202102492 (PMC8564438; doi:10.1002/advs.202102492)
Supplement: Supplementary file 1 — Supporting Information [file ADVS-8-2102492-s001.pdf]

WILEY-VCH

© 2021 Wiley-VCH GmbH

ADVANCED SCIENCE
Open Access

Supporting Information

for *Adv. Sci.,* DOI: 10.1002/advs202102492

Intermediate Phase-Free Process for Methylammonium Lead Iodide Thin Film for High Efficiency Perovskite Solar Cells

Yeonghun Yun, Devthade Vidyasagar, Minho Lee, Oh Yeong Gong, Jina Jung, Hyun-Suk Jung, Dong Hoe Kim, Sangwook Lee**

Supporting Information

Intermediate Phase-Free Process for Methylammonium Lead Iodide Thin Film for High Efficiency Perovskite Solar Cells

Yeonghun Yun, Devthade Vidyasagar, Minho Lee, Oh Yeong Gong, Jina Jung, Hyun-Suk Jung, Dong Hoe Kim, Sangwook Lee**

Table S1. Metrics of perovskite solar cells fabricated using mixed solvents with different ratios (DMF/TMP).

	PCE (%)	J_{SC} (mA cm^{-2})	V_{OC} (V)	FF (%)	HF (%)
0.0 TMP	18.55±0.42	22.18±0.08	1.051±0.017	79.57±0.56	80.24±3.34
0.3 TMP	19.46±0.31	22.25±0.11	1.094±0.007	79.93±0.90	82.30±1.96
1.0 TMP	19.68±0.35	22.10±0.12	1.116±0.004	79.79±1.4	89.39±0.677
3.0 TMP	19.50±0.12	21.93±0.10	1.112±0.005	79.95±0.54	89.51±1.16
5.0 TMP	19.47±0.24	21.91±0.27	1.112±0.002	79.91±1.1	89.76±0.503

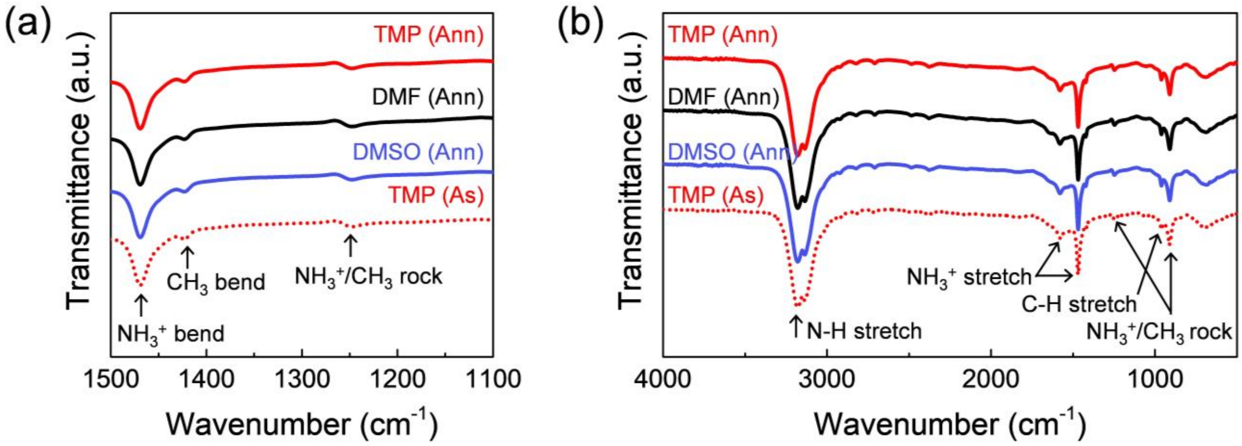

Figure S1. a) Narrow and b) wide range FTIR spectra of annealed (Ann) perovskite films based on each mono-solvent (DMSO, DMF, and TMP) with TMP based as-coated (As) perovskite film.

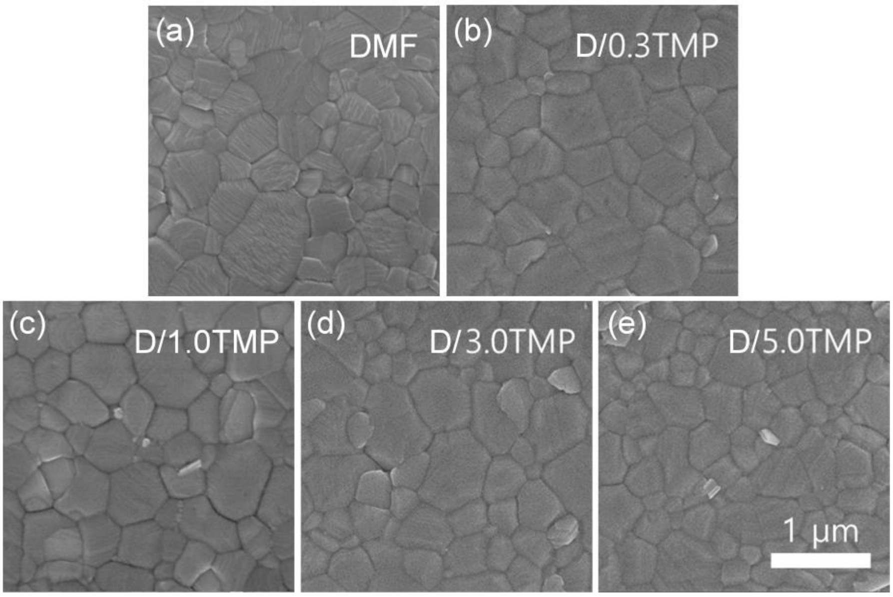

Figure S2. The morphology of PVSK films with varied amount of TMP solvent (here in xTMP, the “x” value represents the mole ratio of TMP with respect to perovskite molar concentration).

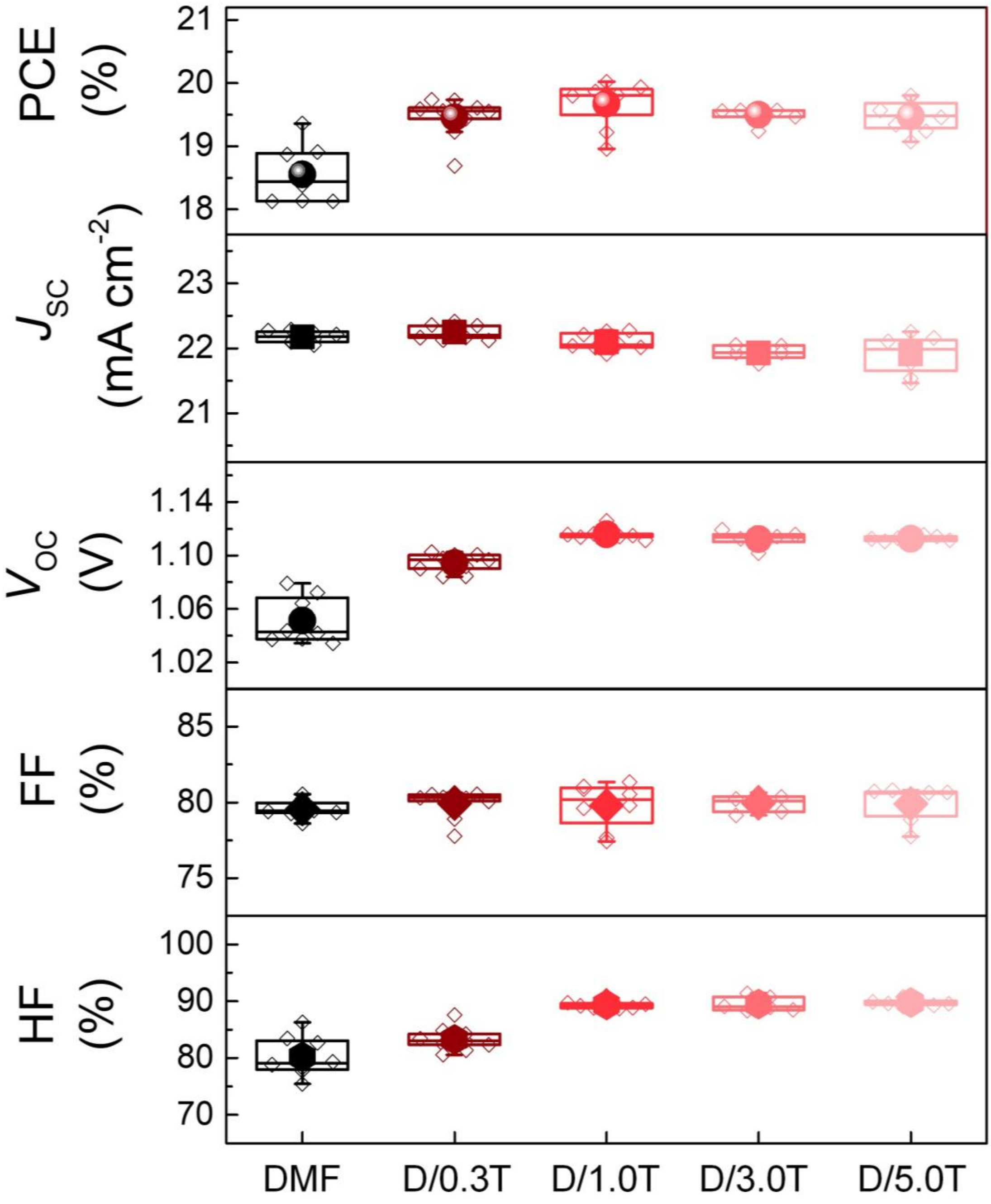

Figure S3. Box charts of the photovoltaic properties obtained from PSCs fabricated with DMF/TMP mixed solvent-based perovskite precursors.

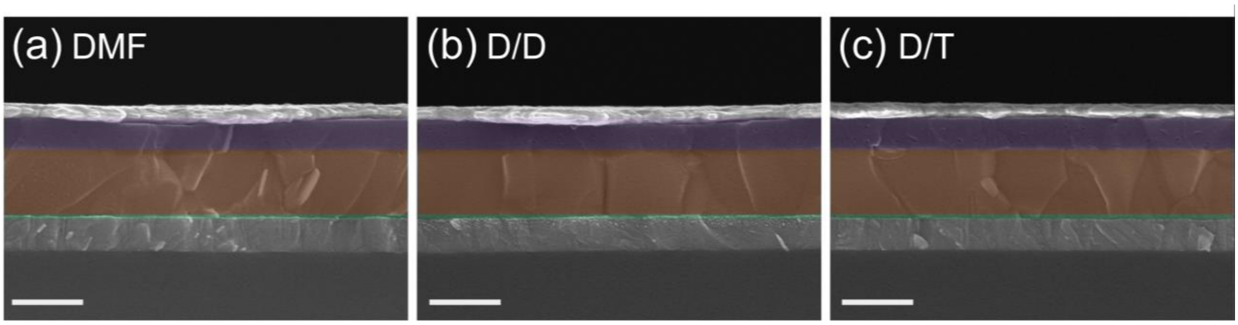

Figure S4. Cross-sectional SEM images of perovskite solar cells obtained from the a) DMF, b) D/D, and c) D/T solvents. Scale bars: 500 nm

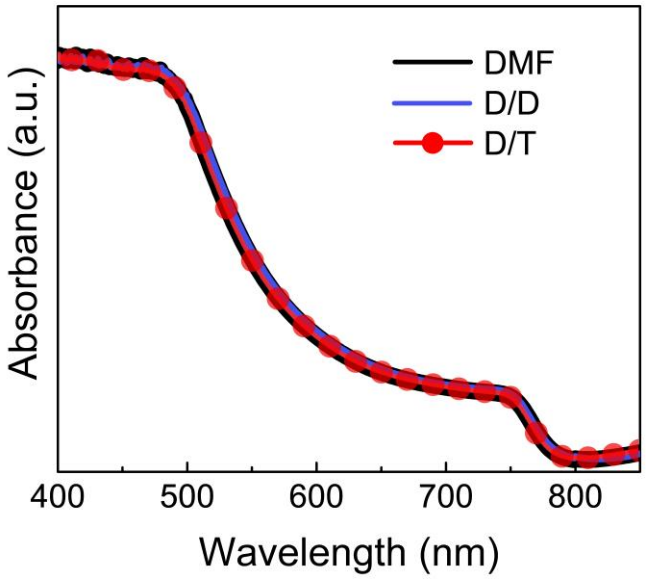

Figure S5. UV–Vis spectra of annealed perovskite films based on the different solvents (DMF, D/D, and D/T).

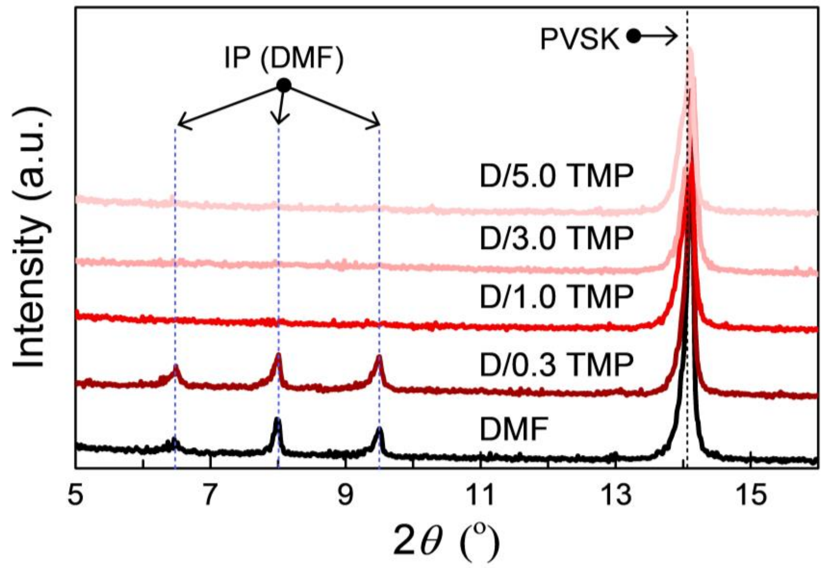

Figure S6. XRD patterns of as-coated films made with DMF/TMP mixed solvents (IP (DMF) means DMF derived intermediate phase).

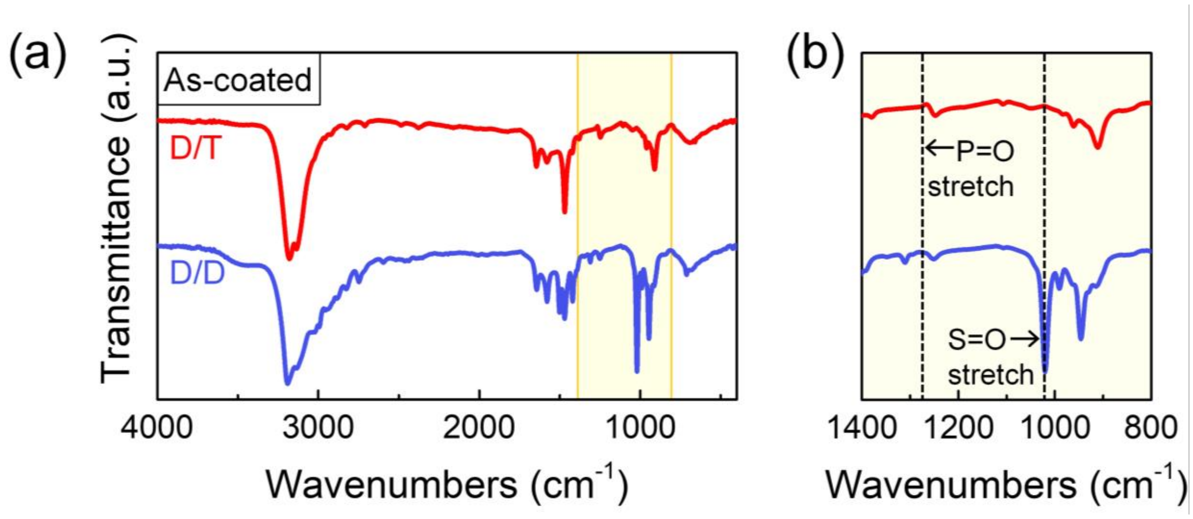

Figure S7. a) FTIR spectra of as-coated perovskite films based on the D/D and D/T solvents. b) Magnified spectra present the region for the S=O and P=O stretching peaks.

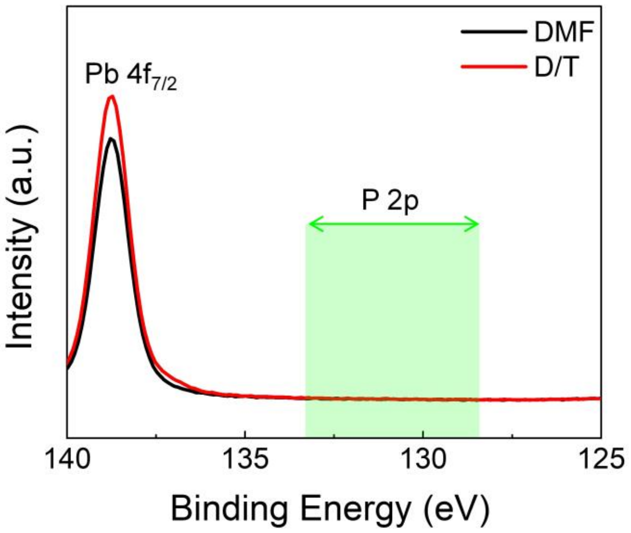

Figure S8. The high-resolution XPS spectra of DMF and D/T films in the binding energy range of 125-140 eV. Phosphorous signal is not detected at all.[1]

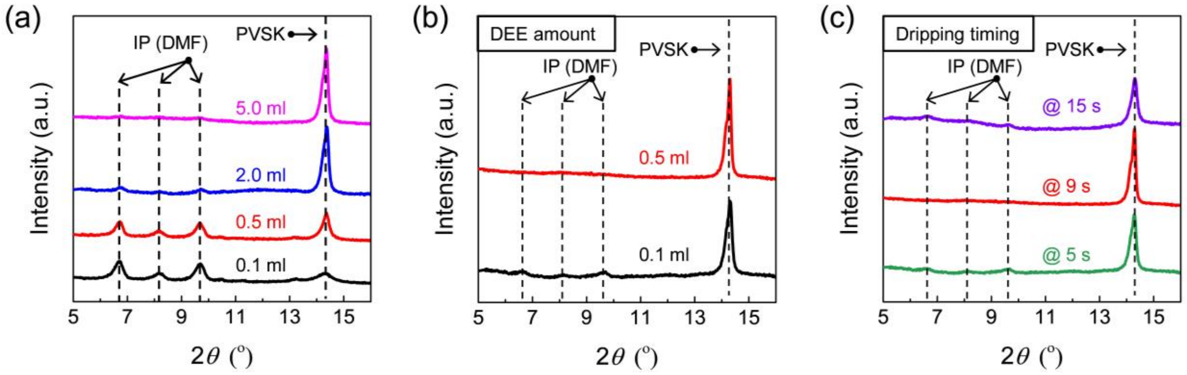

Figure S9. XRD patterns of (a) DMF-based as-coated film with varying the dripping amount of DEE, and D/T-based as-coated films with varying (b) the dripping amount of DEE and (c) the dripping timing.

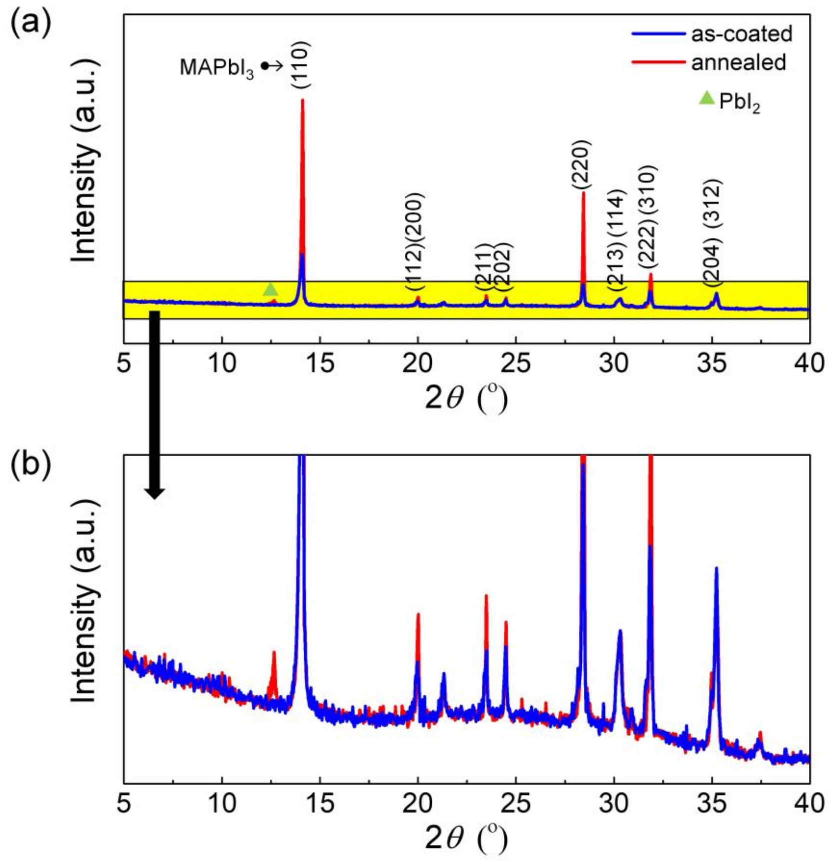

Figure S10. XRD patterns of as-coated and annealed PVSK films based D/T solution in a wide range from 5 to 40 degree.

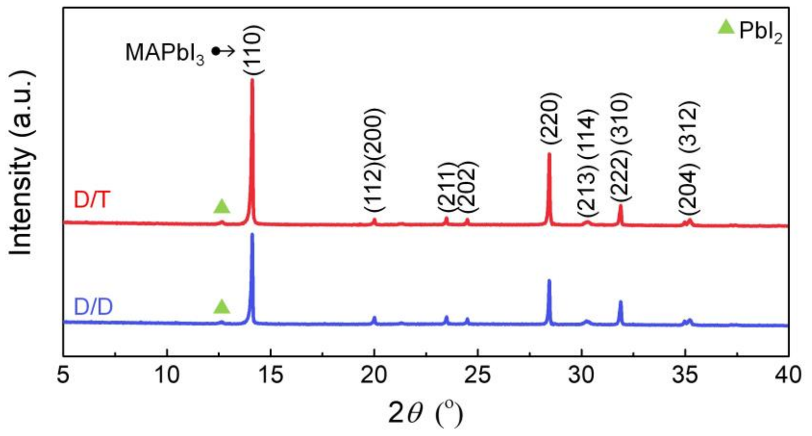

Figure S11. Wide range XRD patterns of annealed perovskite films. The ratio of the peak intensity of (110) to (211) plane is 21.2 and 12.4 for the D/T- and D/D-based film, respectively.

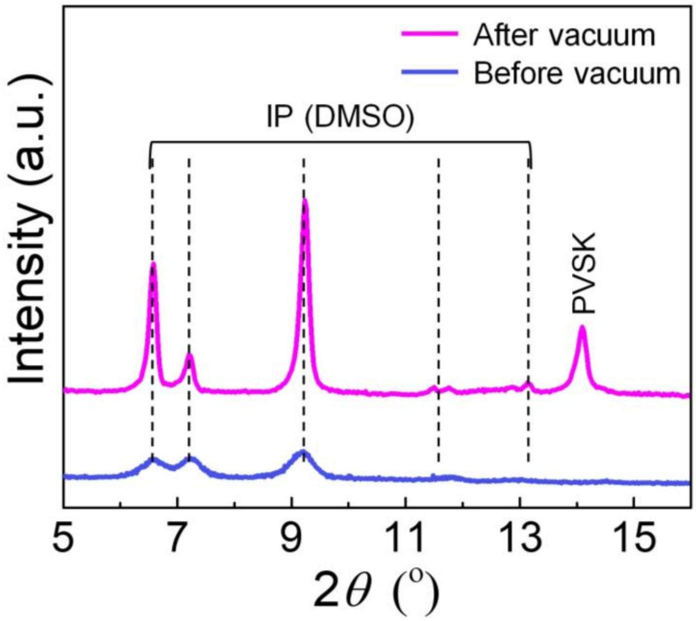

Figure S12. XRD patterns of as-coated DMSO-based films, before and after evacuation in SEM equipment (< 7.2×10^{-7} Torr, for 60 min).

The as-coated D/D film shows large grains (**Figure 5c**), different from the as-coated DMSO film (**Figure 2a**). We attribute this feature to the vacuum condition during SEM imaging. **Figure S12** confirms that the grain size and the crystallinity of the IP (DMSO) is increased after evacuation, evidenced by the decreased full width at half maximum and increased peak area for the intermediate phase. Although the PVSK peak also appears, the main peak is much smaller than that for the intermediate phase. In addition, the perovskite peaks are much broader and smaller than those of the annealed film, though the grain sizes are not different that much. Therefore, it is plausible that the grains shown in **Figure 5c** are mainly intermediate phase, with some portion of perovskite phase. In this regard, although the SEM image for the as-coated D/D film shows an image of grown grains due to the vacuum

condition, it is plausible that the drastic growth of intermediate grains in the D/D film would similarly occur at the beginning of the annealing process.

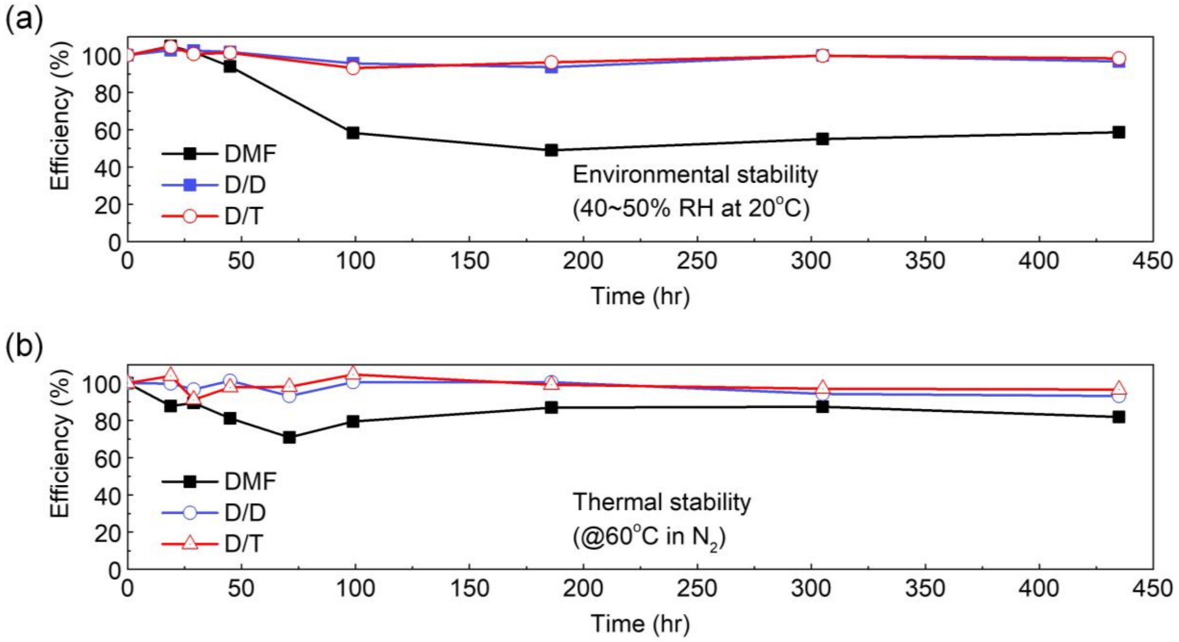

Figure S13. Normalized PCE of unencapsulated devices tracked for 435 h, for humidity and thermal stability. PCE changes (a) in air at a relative humidity (RH) of 40-50% at 20°C, and (b) under nitrogen atmosphere at a temperature of 60°C

References

[1] a) J. Plutnar, Z. Sofer, M. Pumera, *RSC Adv.* **2020**, 10, 36452; b) B. Peng, Y. L. Xu, K. Liu, X. Q. Wang, F. M. Mulder, *Chemelectrochem* **2017**, 4, 2140.
